# Declining abundance of coral reef fish in a World-Heritage-listed marine park

**DOI:** 10.1038/s41598-019-52016-9

**Published:** 2019-10-29

**Authors:** Mathew A. Vanderklift, Russell C. Babcock, Fabio Boschetti, Michael D. E. Haywood, Richard D. Pillans, Damian P. Thomson

**Affiliations:** 1 CSIRO Oceans & Atmosphere, Indian Ocean Marine Research Centre, Crawley, WA 6009 Australia; 2 CSIRO Oceans & Atmosphere, Queensland Biosciences Precinct, St Lucia, QLD 4067 Australia

**Keywords:** Conservation biology, Tropical ecology

## Abstract

One of the most robust metrics for assessing the effectiveness of protected areas is the temporal trend in the abundance of the species they are designed to protect. We surveyed coral-reef fish and living hard coral in and adjacent to a sanctuary zone (SZ: where all forms of fishing are prohibited) in the World Heritage-listed Ningaloo Marine Park during a 10-year period. There were generally more individuals and greater biomass of many fish taxa (especially emperors and parrotfish) in the SZ than the adjacent recreation zone (RZ: where recreational fishing is allowed) — so log response ratios of abundance were usually positive in each year. However, despite this, there was an overall decrease in both SZ and RZ in absolute abundance of some taxa by up to 22% per year, including taxa that are explicitly targeted (emperors) by fishers and taxa that are neither targeted nor frequently captured (most wrasses and butterflyfish). A concomitant decline in the abundance (measured as percentage cover) of living hard coral of 1–7% per year is a plausible explanation for the declining abundance of butterflyfish, but declines in emperors might be more plausibly due to fishing. Our study highlights that information on temporal trends in absolute abundance is needed to assess whether the goals of protected areas are being met: in our study, patterns in absolute abundance across ten years of surveys revealed trends that simple ratios of abundance did not.

## Introduction

Protected areas are a common management tool to protect species and their habitats. Management plans for protected areas frequently set goals that imply (or explicitly state) maintenance or restoration of abundances of species deemed especially worthy of protection (and occasionally to reduce abundances of species deemed unworthy). In the sea, this protection is most commonly implemented as marine parks. One type of marine park is the “no-take” reserve (IUCN Category II), where all forms of fishing are excluded. Numerous authors have advocated “no-take” marine reserves as an effective means of protecting species (e.g.^1,2^), but other authors have argued that such reserves might not be effective under all conditions, and even that narrow focus on “no-take” reserves without considering other management options can be detrimental (e.g.^3,4^). On average, “no-take” reserves tend to host greater relative abundances of fish than fished areas (e.g. see reviews by^5,6^), but this simple metric gives limited insight into whether absolute abundances, or their temporal trends, are adequate to ensure long-term viability. By extension, metrics based on relative abundance provide limited and potentially misleading insights into whether management goals are being met.

Measuring trends in abundance is important if goals include protection or restoration of the abundances of particular taxa^7–9^. In theory (although rarely in practice: e.g.^10^), information on trends should be embedded in an “adaptive approach” in which management actions are implemented to reverse undesirable trends. Understanding which taxa are declining, and where and when this occurs, allows managers to focus on actions that are most likely to arrest or reverse declines. However, reliably detecting trends in abundance presents a number of challenges for ecologists. Accurately estimating abundance is already difficult when individuals are sparsely or patchily distributed, make extensive movements, or are difficult to observe. To accurately quantify the direction and rates of changes in abundance of such taxa through time is an even greater challenge. Yet, such information is central, not only to evaluating the performance of protected areas, but also more generally to decisions about which species to protect, and how to protect them.

Abundance can be quantified in different ways, including counts of the number of individuals, counts of individuals per unit area (density), and biomass (a measure which is frequently used to estimate the abundance of fish). Each of these can yield different estimates of trend. Here, we evaluate trends in the abundance (measured as density and biomass) of fish in the Ningaloo Marine Park (NMP), located within the Ningaloo Coast World Heritage Area, north-western Australia. Several previous studies have indicated that sanctuary zones (SZ, equivalent to IUCN Category II) within the park contain higher abundances of some fishes than zones which allow recreational fishing^11–13^, but our ability to detect long-term trends is hampered, because these studies have used different techniques (visual census and baited video), have been carried out at different locations, or use relative abundance. We surveyed fish assemblages of shallow reefs in the majority of years from 2007–2016; we then sought to determine whether our surveys yielded evidence of increasing or decreasing trends during that period, and if so, to quantify what the trend was, and if it varied between management zones designed to control fishing.

## Methods

### Study area and survey design

Our study was conducted on shallow reef flats within and adjacent to the Mandu Sanctuary Zone (SZ: 1,185 ha) in the Ningaloo Marine Park (NMP: 22°S, 113°E), which was established in May 1989. The coral reefs in NMP are mainly fringing reefs located 0.2–2 km from the shore. The climate is arid and there is little runoff from land. The reef flat is ~150 m wide, generally submerged even at low tide, and in the Mandu region is typically dominated by the tabulate coral *Acropora spicifera*^14^.

We surveyed eight sites in the SZ, and eight sites in the adjoining Recreation Zone (RZ): the same sites were surveyed in each year. Commercial fishing is not permitted in SZs or RZs in the NMP. Recreational angling is permitted in RZs and is the main type of fishing within the reef flat habitat in NMP; spearfishing is not permitted anywhere in the vicinity of Mandu, including RZs. Although human population density along the coast is low, the area is very popular with tourists and fishing effort can be high; for example, a survey in 1998–99 recorded >85,000 fisher days in the Ningaloo Marine Park during 12 months^15,16^.

Our surveys were first conducted in 2007 (18 years after the NMP was established), and again in 2009, 2010, and each year from 2012 to 2016. Using the Sample 3.03 extension for ArcView 3.3^17^, a 200-m grid was overlaid across the study area, from which potential sites were selected by generating a single random point within each grid cell. Sites from among the randomly-generated points were then selected to ensure a balanced distribution of sites within the SZ (eight sites), and in the adjacent RZ to the north and south (eight sites).

### Survey methods

Fish were surveyed by visual census along three haphazardly-selected 25 × 5 m transects at each site in each survey. All individuals were included except small (<4 cm) or cryptic species that are poorly surveyed using this method. The species identity, number and size (estimated to nearest 5 cm) of fish were recorded. Surveys were not always carried out in the same month each year. In most years a single survey was conducted, but in 2014 and 2015 we conducted two surveys per year. We did this to test if the time of year might influence the estimates. We surveyed in March and November in each of those two years, and tested for differences using mixed effects orthogonal ANOVA on log-transformed data. In 23 of 24 cases there was no statistically significant difference at P > 0.07. In one case (counts of *Lethrinus atkinsoni*), there was a small but statistically significant at P = 0.047. However, given the sequence that months were surveyed in different years (April, November, June, November, May, March, November, March, November), any seasonal variation – should it exist – would not explain temporal trends across the ten years surveyed, but would rather tend to increase variability and dampen the pattern.

The entire 25-m transect was photographed at approximately 0.5-m intervals using a digital camera, facing directly downwards at a distance of approximately 0.5-m above the substrate. Thirty-two photographs per transect were selected, and the benthos immediately beneath five (2007 and 2008) or six (2009 to 2016) fixed points per photograph was recorded (yielding 160 and 192 observations per transect respectively), using the software TransectMeasure (www.seagis.com.au).

Fish counts and sizes were converted to biomass (kg), using the formula B = a × L^b^, where B is biomass, L is total length estimated by divers, and a and b are constants taken from the average values for the relevant family for which conversions using total length were present in FishBase (www.fishbase.org).

### Statistical analyses

In order to focus analyses on taxa that were reliably surveyed, and so would yield the most robust estimates, analyses were restricted to families that were the highest ranked in terms of their contribution to overall biomass, and were recorded on more than 25% of transects. The species included were those that comprised the highest biomass contribution for each of the families selected.

We analysed patterns in fish abundance in two ways. First, we tested whether trends over time tended to differ from zero, and if so whether the trend was an increase or a decrease, and whether the trend differed between SZ and RZ. Second, we used log response ratios to evaluate whether taxa tended to be more abundant in a particular zone in each year.

To test whether trends in abundance tended to differ from zero, we estimated the annual rate of change (**u**) in (log-transformed) counts and biomasses using Multivariate Auto-Regressive State-Space modelling, using the MARSS package^18^ in the statistical software R^19^. We used means calculated from all transects surveyed in each year (including when there were multiple surveys within a year). For years in which surveys were not conducted we allocated a missing data value. MARSS provides confidence intervals for estimates of **u**, with an assumption that the (logarithm of the) data is suitably approximated by a linear process with Gaussian errors. To minimise bias in the analysis, and because we had no *a priori* basis on which to estimate process and observation errors, these were not defined and so were computed together with the estimate of **u**. Similarly, no covariance in process and observation errors between SZ and RZ was set *a priori*. (A discussion of the treatment of process and observation errors in MARSS can be found in section 4.2 of Holmes *et al*.^18^)

To assess the statistical significance of **u** we employed two complementary bootstrapping approaches^20^. In the first approach, we used the parametric bootstrapping option in the function MARSSparamCIs to compute the 95% confidence intervals of **u**. If 95% confidence intervals of **u** did not overlap zero, we inferred support for a statistically-significant rate of change. In the second approach, we used a method described in Unsworth *et al*.^21^ to test the null hypothesis of no rate of change. Both bootstrapping approaches are based on first generating a large number (1000) of surrogate datasets, computing a MARSS model for each, and then computing confidence intervals and p-values from the distribution of models. The core difference between the two approaches lies in how the surrogate time series are generated. In the first approach, the original data is used to generate a set of datasets that share the same trend **u**, but that differ in the distribution of process and observation errors (see sections 3.6 and 5.12 in^18^). In the second approach, the year label for each original observation is randomly re-ordered and the model is re-calculated for each dataset. In this approach the observations are conserved but the trend is not. As a result, in the first method the bootstrapping method works at the resolution of yearly averages while in the second it works at the resolution of observations. Here, we use both methods on the basis that their agreement provides a stronger evidence for the significance of the result than either in isolation. When they yielded different results, we inferred that some uncertainty exists about whether there is indeed a trend.

To assess support for the competing hypotheses that SZ and RZ (a) have different trends or (b) share a common trend, we used a variant of the second bootstrapping method described above, in which the location of the observations (i.e. in the SZ or the RZ) — rather than their date — was randomly re-ordered to generate a set of surrogate datasets. If the model based on the observations cannot be distinguished from the distribution of models obtained from these surrogate datasets, it follows that the null hypothesis that SZ and RZ share a common trend **u** should not be rejected. This was tested by comparing the distribution of **u** for each of SZ and RZ against the overall **u** of the pooled data (i.e. a separate test for each).

Log response ratios were calculated as: *LRR* = $ln(\overline{SZ})$ − $\mathrm{ln}(\overline{RZ})$, where $\overline{SZ}$ is the mean observation in the Sanctuary Zone, and $\overline{RZ}$ is the mean observation in the adjacent Recreation Zone. Separate analyses were conducted for each taxon in each survey, and 95% confidence intervals were calculated. Analyses were done using the metafor package in the statistical software R^22^.

## Results

**Figure 1:**
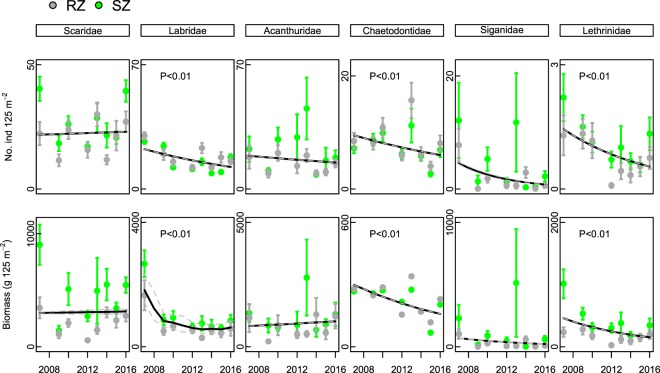
Mean counts and biomasses (±SE) of six families of fishes for SZ (green symbols) and RZ (red symbols) and estimated trend from MARSS model (black line).

There was no support for a difference in trends between SZ and RZ in densities (counts) of any family (with the single exception of Labridae, for which there was equivocal support for a difference), so we considered the combined trends (although trends for each zone are also provided for completeness: Table 1). In the pooled data, there were significantly decreasing trends (95% CI not overlapping zero and statistical significance yielded by bootstrapping) in counts of wrasses (Labridae: 4.7–17.1% decline per year), butterflyfish (Chaetodontidae: 0.0–18.1%), and emperors (Lethrinidae: 7.5–22.5%) (Table 1, Fig. 1). Comparisons of SZ and RZ also yielded little support for the hypothesis of a difference between SZ and RZ for any species, aside from equivocal support for the rabbitfish *Siganus fuscescens*, and so pooled trends were examined (Table 1). From the pooled trend, counts of the wrasse *Coris aygula* (6.7–22.0%), the emperor *Lethrinus atkinsoni* (7.2–20.9%) and the butterflyfish *Chaetodon plebeius* (3.5–20.3%) all declined significantly during the survey period (Table 2).

**Table 1:** Estimated annual trend **u**, and upper and lower 95% confidence intervals generated by MARSS using annual mean counts for (a) each family and (b) each species.

	Pooled				SZ					RZ				
	u	Lower CI	Upper CI	p-Time	u	Lower CI	Upper CI	p-Time	p-Zone	u	Lower CI	Upper CI	p-Time	p-Zone
**(a)**														
Scaridae	−0.037	−0.108	0.033	0.014	−0.05	−0.11	0.007	0.015	0.194	−0.019	−0.107	0.068	0.261	0.117
Labridae	−**0.108**	−**0.171**	−**0.047**	**0.001**	−**0.124**	−**0.175**	−**0.074**	**0.001**	**0.034**	−**0.095**	−**0.169**	−**0.021**	**0.001**	0.087
Acanthuridae	−0.065	−0.183	0.056	0.029	−0.062	−0.212	0.095	0.134	0.4	−0.073	−0.156	0.006	**0.015**	0.41
Chaetodontidae	−**0.09**	−**0.181**	**0**	**0.001**	−**0.102**	−**0.2**	−**0.002**	**0.001**	0.239	−0.08	−0.177	0.014	**0.001**	0.239
Siganidae	−0.238	−0.492	0.015	0.005	−0.278	−0.577	0.007	**0.006**	0.286	−0.227	−0.634	0.234	**0.024**	0.452
Lethrinidae	−**0.15**	−**0.225**	−**0.075**	**0.001**	−**0.14**	−**0.181**	−**0.094**	**0.002**	0.404	−0.167	−0.353	0.022	**0.01**	0.287
**(b)**														
*Chlorurus sordidus*	−0.005	−0.081	0.063	0.381	−0.012	−0.067	0.045	0.352	0.327	−0.005	−0.12	0.106	0.447	0.496
*Acanthurus triostegus*	−0.071	−0.231	0.071	0.058	−0.069	−0.238	0.106	0.141	0.45	−0.075	−0.179	0.02	**0.035**	0.447
*Coris aygula*	−**0.142**	−**0.22**	−**0.067**	**0.001**	−**0.12**	−**0.198**	−**0.041**	**0.001**	0.199	−**0.176**	−**0.254**	−**0.097**	**0.001**	0.15
*Siganus nebulosus*	−0.261	−0.521	0.003	0.003	−0.222	−0.5	0.049	**0.044**	0.284	−**0.433**	−**0.711**	−**0.162**	**0.001**	**0.024**
*Lethrinus atkinsoni*	−**0.142**	−**0.209**	−**0.072**	**0.001**	−**0.138**	−**0.174**	−**0.096**	**0.003**	0.444	−0.15	−0.317	0.013	**0.011**	0.386
*Chaetodon plebeius*	−**0.12**	−**0.203**	−**0.035**	**0.001**	−**0.129**	−**0.21**	−**0.049**	**0.001**	0.359	−**0.111**	−**0.199**	−**0.023**	**0.001**	0.278

Bold font indicates estimates with 95% confidence intervals not overlapping zero, and so robust evidence of a declining trend.

**Table 2:** Estimated annual trend **u**, and upper and lower 95% confidence intervals generated by MARSS using annual mean biomass for (a) each family and (b) each species.

	Pooled				SZ					RZ				
	u	Lower CI	Upper CI	p-Time	u	Lower CI	Upper CI	p-Time	p-Zone	u	Lower CI	Upper CI	p-Time	p-Zone
**(a)**														
Scaridae	−0.038	−0.164	0.082	0.123	−0.043	−0.178	0.098	0.192	0.408	−0.027	−0.164	0.114	0.273	0.307
Labridae	−**0.151**	−**0.214**	−**0.086**	**0.001**	**-0.182**	−**0.315**	−**0.065**	**0.001**	0.204	−**0.144**	−**0.225**	−**0.06**	**0.002**	0.253
Acanthuridae	−0.019	−0.114	0.069	0.344	−0.036	−0.154	0.08	0.297	0.409	0.001	−0.131	0.151	0.458	0.239
Chaetodontidae	−**0.11**	−**0.185**	−**0.038**	**0.001**	−**0.127**	−**0.208**	−**0.038**	**0.001**	0.163	−**0.097**	−**0.174**	−**0.021**	**0.002**	0.192
Siganidae	−0.173	−0.476	0.151	0.082	−0.16	−0.498	0.159	0.174	0.278	−**0.378**	−**0.769**	−**0.025**	**0.005**	**0.035**
Lethrinidae	−**0.166**	−**0.226**	−**0.106**	**0.001**	−**0.191**	−**0.254**	−**0.128**	**0.001**	0.251	−0.112	−0.275	0.05	**0.033**	**0.026**
**(b)**														
*Chlorurus sordidus*	−0.016	−0.144	0.124	0.315	−0.031	−0.207	0.142	0.261	0.199	0.007	-0.124	0.145	0.374	0.161
*Acanthurus triostegus*	−0.133	−0.296	0.035	**0.013**	−0.089	−0.273	0.101	0.115	0.143	−**0.201**	−**0.342**	−**0.072**	**0.004**	**0.028**
*Coris aygula*	−**0.159**	−**0.254**	**-0.071**	**0.001**	−**0.168**	−**0.261**	−**0.084**	**0.003**	0.48	−**0.146**	−**0.229**	−**0.06**	**0.024**	0.307
*Siganus nebulosus*	−0.181	−0.502	0.131	0.086	−0.157	−0.503	0.148	0.178	0.24	**−0.388**	−**0.784**	−**0.02**	**0.001**	**0.025**
*Lethrinus atkinsoni*	−**0.161**	−**0.21**	−**0.108**	**0.001**	−**0.185**	−**0.236**	−**0.131**	**0.001**	0.257	−0.104	−0.261	0.063	0.046	**0.026**
*Chaetodon plebeius*	−**0.14**	−**0.187**	−**0.088**	**0.001**	−**0.159**	−**0.278**	−**0.032**	**0.001**	0.194	−**0.121**	−**0.177**	−**0.065**	**0.001**	0.159

Bold font indicates estimates with 95% confidence intervals not overlapping zero, and so robust evidence of a declining trend.

For biomass, there was again limited support for the hypothesis that trends differed between SZ and RZ, with only equivocal support for emperors and rabbitfish (Table 2, Fig. 1). Trends in biomass of rabbitfish (Siganidae) were highly influenced by a few extreme biomass records. From the pooled data, there were overall declining trends shared across zones in biomass of wrasses (Labridae: 8.6–21.4%), butterflyfish (Chaetodontidae: 3.8–18.5%) and emperors (Lethrinidae: 10.6–22.6%) (Table 2). Similarly, there were overall decreasing trends in biomass of the wrasse *Coris aygula* (7.1–25.4%), the butterflyfish *Chaetodon plebeius* (8.8–18.7%) and the emperor *Lethrinus atkinsoni* (10.8–21.0%) (Table 2). Notably, there was no evidence for an increasing trend in either counts or biomass of any taxon.

There was an overall decreasing trend in percentage cover of living hard coral (a decline of 1.4–7.5% per year), but no statistically-significant overall trends in the percentage cover of algae or abiotic substrate (Table 3). There was some support for the hypothesis that the percentage cover of living coral declined strongest in the SZ (P = 0.01 and P = 0.05, Table 3).

**Table 3:** Estimated annual trend **u**, with upper and lower 95% confidence intervals generated by MARSS using percentage cover of three benthic habitat cover classes, and rugosity. Bold font indicates estimates with 95% confidence intervals not overlapping zero, and so a robust evidence of a declining trend. Pooled = Sanctuary and Recreational Zone combined.

	Pooled				SZ					RZ				
	u	Lower CI	Upper CI	p-Time	u	Lower CI	Upper CI	p-Time	p-Zone	u	Lower CI	Upper CI	p-Time	p-Zone
Hard coral (%)	**−0.046**	**−0.075**	**−0.014**	**0.005**	−0.083	−0.214	0.058	0.001	**0.015**	−0.019	−0.081	0.031	0.202	0.051
Algae (%)	0.026	−0.015	0.069	0.002	0.032	−0.046	0.103	0	0.098	0.019	−0.042	0.07	0.073	0.104
Abiotic (%)	−0.043	−0.171	0.099	0.049	−0.051	−0.245	0.162	0.01	0.243	−0.039	−0.198	0.148	0.153	0.423
Rugosity	0.019	−0.001	0.038	0	0.015	−0.009	0.037	0	0.099	0.021	0.002	0.036	0	0.217

**Figure 2:**
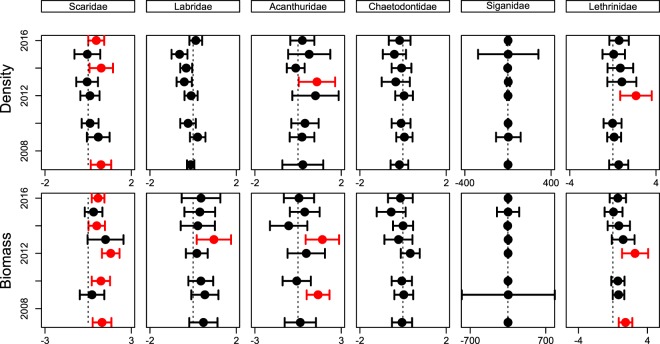
Log response ratios (±95% CI) of six families of fishes calculated from counts (top row) and biomasses (bottom row). Red coloured values indicate statistically significant higher values inside Sanctuary Zones. Dotted line runs through zero (if the 95% CI cross the dotted line, it indicates that observations from SZ and RZ are identical).

For each family, LRR calculated from both counts and biomass tended to fluctuate from year to year during the study. Robust evidence of higher counts in the SZ (i.e. 95% CI not overlapping zero) in more than one year was present only for parrotfish (2007, 2014 and 2016) (Fig. 2). The pattern tended to be more pronounced for biomass, with robust evidence for higher biomass in the SZ in more than one year for emperors (2007, 2012), parrotfish (2007, 2010, 2012, 2014, 2016), and surgeonfish (2009, 2013) (Fig. 2).

**Figure 3:**
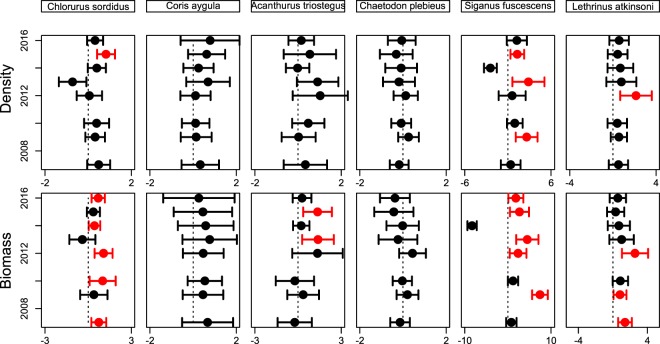
Log response ratios (±95% CI) of six species of fishes calculated from counts (top row) and biomasses (bottom row). Positive values indicate higher values inside Sanctuary Zones. Dotted line runs through zero (which would reflect observations from SZ and RZ would be identical).

Similar patterns were observed for the main species analysed (Fig. 3). Analyses based on counts yielded variable results among years, while analyses based on biomass yielded higher biomasses in SZ for *Chlorurus sordidus* (2007, 2010, 2012, 2014, 2016), *Acanthurus triostegus* (2013, 2015), and *Lethrinus atkinsoni* (2007, 2009, 2012). Results for *Siganus fuscescens* were variable, with surveys in some years yielding higher biomass in the SZ (2009, 2012, 2013, 2015, 2016) but 2014 yielding higher biomass in the RZ.

## Discussion

Ten years of observations in and around the Mandu Sanctuary Zone revealed a consistent decline in abundance for multiple taxa in both the protected sanctuary zone (IUCN Category II), and an adjacent zone open to recreational fishing. This decline occurred despite the abundance of some taxa, like emperors, tending to be higher in the SZ (especially when using biomass). The abundance of butterflyfish and wrasse were roughly similar in the SZ and RZ, but declined significantly during the survey. In contrast, parrotfish (counts and biomass) and surgeonfish (biomass only) tended to be more abundant in the SZ but their abundances did not show any overall trend during the study.

The global consensus on the effectiveness of “no-take” marine reserves is that they are generally successful in achieving conservation outcomes^6,23^ — if these are defined by the simple metric of whether abundances of some species are higher inside protected area boundaries than outside them. However, the simple metric of comparing abundance within a protected area with that in adjacent fished areas can obscure temporal dynamics — in fact, our data show that the most typically used measure of effect size, the log response ratio, can vary little even as overall abundances decline. In our study, we found significant declines in abundances (as both counts and biomass) of several taxa (e.g. emperors, butterflyfish) without corresponding changes in effect size. These results highlight that effect sizes should be used cautiously (or perhaps not at all) when evaluating whether protected areas meet objectives that are related to conservation of species^24^. In the Ningaloo Marine Park, specific objectives for fish are that there is no loss of abundance in highly-protected zones (including SZs) as a result of human activities^25^. Our surveys cannot determine the causes of declines (although we set out some plausible hypotheses below), but it seems likely that the Mandu SZ is not meeting this objective, although a more limited definition of success — i.e. higher abundance inside the SZ — is being achieved for some taxa. These results highlight how undue focus on effect sizes alone could lead to substantial declines being overlooked.

A more complete understanding of the effectiveness of protected areas demands careful compilation of observations over long periods of time to allow trends to be revealed. In some places, such studies have shown that abundances of many taxa increase in marine reserves over time (e.g.^26^), while in others studies have shown long-term decreases: for example, Bornt *et al*.^27^ showed a decline in several taxa (including emperors) at the Houtman Abrolhos Islands (off the coast of western Australia) over a span of 9 years, while Russ & Leahy^28^ showed that declining abundances of butterflyfish spanning protected areas and adjacent fished areas in the Philippines could occur rapidly in response to a reduction in the abundance of living hard coral.

In our study, abundances generally declined at similar rates in both the SZ and RZ. There are several possible causes for these declines, and it seems plausible—even likely—that the causes vary among taxa. We can develop hypotheses about the possible causes by drawing on knowledge of the biology and ecology of the taxa. The declining abundance of some species (such as emperors) is plausibly due to fishing. Even relatively low rates of mortality due to fishing might tend to decrease abundance if recruitment and growth rates are low. Although the human population in the region is small, recreational fishing effort can be high, and emperors are sought after by fishers^15,29^. An additional (and complementary) plausible explanation is that the reef within Mandu SZ is nested in a larger contiguous area of fringing reef; the movement ranges of some taxa—including emperors^30,31^—is likely to result in many individuals moving outside the SZ, resulting in enough fishing mortality to produce a decline. Although *Lethrinus atkinsoni* (the most abundant emperor on the reef flat) does not feature strongly in catch statistics, it is frequently caught by campers on the coast, who do not tend to be included in boat ramp surveys, and is also used as bait for larger fish (authors’ personal observations).

There is evidence that this trend may have existed for some time as our estimates of counts and biomass of emperors at Mandu in 2007 (our first observations) were somewhat lower than those from earlier studies^11,13^. The most commonly landed emperor at Ningaloo, *Lethrinus nebulosus*, was only observed on our transects twice since 2010 (it was recorded during each survey from 2007 to 2010); it would have been desirable to analyse trends in abundance of this species, but the very low numbers of individuals observed precluded such analyses. Nevertheless, our observations are consistent with modelling of the recreational fishery for *L. nebulosus* in 2007, which indicated that the population of *L. nebulosus* on Ningaloo was at around half of unfished biomass and that, if there were no changes to bag or size limits, the population of *L. nebulosus* would likely continue to decline^32^. It is also consistent with a study that indicated there was localised overfishing in the region^33^.

Fishing is implausible as an explanation for the observed decrease in abundance of butterflyfish, because they are not captured by recreational or commercial fishers within the marine park. Instead, a more plausible explanation is a change in habitat—particularly the living hard corals that are a food source for several of the species that inhabit the reef flat at Mandu. Butterflyfish tend to be strongly associated with living hard coral, which declined during our survey period, likely because of mortality during the coral bleaching episodes experienced on the western Australian coast in 2011 and 2013^34,35^. The potential decline in cover of living hard coral is not likely to be a cause of declines in abundance of wrasses or emperors, for which strong associations with living hard coral are not present.

Potential causes of declines in the abundance of the large wrasse *Coris aygula* are unclear, because there is little evidence that they are caught by recreational fishers, either from published catch statistics or from our own observations, and they do not show particularly strong affinity for living coral. In some other parts of its range it is present in higher abundance within marine reserves^36^.

There were also some taxa that did not show evidence for a temporal trend, either declining or increasing, during the survey period — including parrotfishes and surgeonfishes. (Trends for rabbitfishes are likely influenced by a few extreme values and might not reflect meaningful change.) Together, these patterns demonstrate that declines were not ubiquitous across all taxa, but were restricted to some taxa — likely those with traits that render them particularly susceptible.

Our surveys were not designed to identify the causes of trends, and we can only propose plausible mechanisms based on our knowledge of the biology and ecology of the species involved. Nevertheless, the surveys demonstrate that declines in abundance of desired species can occur, even in protected areas with robust management. In order for the goals of protected areas to be met, information on temporal trends in abundance of desired taxa is needed, and management arrangements need to have the flexibility to incorporate information (including tests of hypotheses about causes) and implement actions to halt or reverse the trends.
